# Comprehensive genomic analyses of *Vigna unguiculata* provide insights into population differentiation and the genetic basis of key agricultural traits

**DOI:** 10.1111/pbi.14047

**Published:** 2023-04-04

**Authors:** Lei Pan, Minghui Liu, Yan Kang, Xiang Mei, Gege Hu, Chun Bao, Yu Zheng, Huixia Zhao, Chanyou Chen, Nian Wang

**Affiliations:** ^1^ Hubei Province Engineering Research Center of Legume Plants, School of Life Sciences Jianghan University Wuhan China; ^2^ College of Horticulture and Forestry Sciences Huazhong Agricultural University Wuhan China

**Keywords:** *Vigna unguiculata*, chromosome‐scale genome, genome‐wide association study (GWAS), natural and artificial selections, pod length, pod width

## Abstract

*Vigna unguiculata* is an important legume crop worldwide. The subsp. *sesquipedalis* and *unguiculata* are the two major types grown; the former is mainly grown in Asia to produce fresh pods, while the latter is mainly grown in Africa to produce seeds. Here, a chromosome‐scale genome for subsp. *sesquipedalis* was generated by combining high‐fidelity (HiFi) long‐read sequencing with high‐throughput chromosome conformation capture (Hi‐C) technology. The genome size for all contigs and N50 were 594 and 18.5 Mb, respectively. The Hi‐C interaction map helped cluster 91% of the contigs into 11 chromosomes. Genome comparisons between subsp. *sesquipedalis* and *unguiculata* revealed extensive genomic variations, and some variations resulted in gene loss. A germplasm panel with 315 accessions of *V. unguiculata* was resequenced, and a genomic variation map was constructed. Population structure and phylogenetic analyses suggested that subsp. *sesquipedalis* originated from subsp. *unguiculata*. Highly differentiated genomic regions were also identified, and a number of genes functionally enriched in adaptations were located in these regions. Two traits, pod length (PL) and pod width (PW), were observed for this germplasm, and genome‐wide association analysis of these traits was performed. The quantitative trait loci (QTLs) for these two traits were identified, and their candidate genes were uncovered. Interestingly, genomic regions of PL QTLs also showed strong signals of artificial selection. Taken together, the results of this study provide novel insights into the population differentiation and genetic basis of key agricultural traits in *V. unguiculata*.

## Introduction

*Vigna unguiculata* [L.] Walp. (2*n* = 2*x* = 22), a member of the Fabaceae family, is an annual tropical or subtropical herb grown for human food and animal feed as a green manure, hay, or silage (Kouakou *et al*., 2022; Singh *et al*., 1997; Timko and Singh, 2008). This legume was used in ancient Africa and Asia. *V. unguiculata* is indigenous to tropical Africa, where wild types still occur, and its domestication is thought to have occurred in west‐central or south‐central Africa, in the region from Nigeria to Zaire (Ehlers and Hall, 1997). It is widely distributed in tropical and subtropical regions of sub‐Saharan Africa (SSA) (Dhaliwal *et al*., 2020). *V. unguiculata* is also cultivated in Latin America, Southeast Asia, the Mediterranean Basin and the United States (FAOSTAT, www.fao.org). The Latin name *Unguiculata* refers to the small stalks of their petals, meaning “with a small claw” or “with a small nail”. Its common name, “cowpea”, is based on its use as fodder for cows (Timko and Singh, 2008). As a member within the subspecies *unguiculata*, cultivated cowpea can be classified into five cultivar groups Unguiculata, Sesquipedalis, Textilis, Biflora and Melanophthalmus (Pasquet, 1998, 2000), and they are also considered five subspecies.

Molecular evidence indicated that cultivated *V. unguiculata* was originally domesticated in both East and West Africa and then spread to every continent except Antarctica (Chen *et al*., 2017; Herniter *et al*., 2020; Xiong *et al*., 2016). Generally, the subspecies *unguiculata* and *sesquipedalis* cultivar groups are the two major cultivated types (Ehlers and Hall, 1997). The subspecies *unguiculata* is mainly grown in Africa for the production of seeds, while the subspecies *sesquipedalis* group (also known as yard long bean) is mainly grown in Asia, especially in China, for the production of fresh pods. According to the molecular evidence, it is easy to predict that subsp. *sesquipedalis* originated from subsp. *unguiculata* (Herniter *et al*., 2020). Regarding the different growth conditions in Africa and China and different breeding aims for the subsp. *sesquipedalis* and *unguiculata*, there is extensive variation between these two subspecies. Although a number of studies have focused on investigating the domestication of *V. unguiculata*, limited knowledge has been shared regarding the difference between these two subspecies, especially at the genomic level.

The rapid growth of plant genome sequencing has given rise to significant increases in publicly available genomic resources over the past two decades (Marks *et al*., 2021). Within the *Vigna* crops, the chromosome‐scale genome assembly of several legumes has been completed. The total estimated *V. radiata* var. *radiata* genome was 421 Mb with 22 427 protein‐coding genes (Kang *et al*., 2014). For Indian black gram [*Vigna mungo* (L.) Hepper var. *mungo*], a long‐read‐based draft genome sequence was assembled with a cumulative size of ∼454.4 Mb and an N50 size of 42.88 Mb (Ambreen *et al*., 2022). The total scaffold sequences amounted to 466.7 Mb (N50 = 1.29 Mb), and 34 183 protein‐coding genes were predicted for adzuki bean (*V. angularis*) (Yang *et al*., 2015). Based on PacBio long‐read data and a high‐throughput chromosome conformation capture (Hi‐C) interaction map, an improved rice bean (*V. umbellata*) genome of 475.64 Mb containing 26 736 protein‐coding genes with an N50 of 18.26 Mb was obtained (Guan *et al*., 2022). For *V. unguiculata* (cowpea), a 519.4 Mb sequence scaffold was generated with an N50 of 16.4 Mb (Lonardi *et al*., 2019). The subsp. *unguiculata* line IT97K‐49‐35 (assigned IT97K subsequently) was used as plant material for genome sequencing and assembly. Previously, *V. unguiculata* subsp. *sesquipedalis* var. Xiabao II (also known as A147) was used for *de novo* genome assembly with short‐read sequencing (Xia *et al*., 2019). The draft contig assembly for Xiabao II was 549.8 Mb, and its contig N50 size was only 15.2 kb. Thus, as a widely grown vegetable crop in China and Asia, there is a lack of high‐quality chromosome‐scale references for subsp. *sesquipedalis*. Moreover, there is also a lack of genome comparison between subsp. *sesquipedalis* and *unguiculata*.

Benefiting from low‐cost and high‐throughput next‐generation sequencing technology, whole‐genome resequencing (WGS) of a large panel of germplasms has become feasible (Yao *et al*., 2020). The genome‐wide association study (GWAS) approach is a popular approach for trait quantitative trait locus (QTL) identification with increased resolution. Combining WGS and GWAS for complete genetic populations or diverse germplasm sets has been successfully carried out for several legume crop species. For example, QTL mapping of flowering time control, seed development and pod dehiscence was performed in pigeon pea (Varshney *et al*., 2017); drought tolerance‐related traits in chickpea (Kale *et al*., 2015); resistance to early leaf spot (ELS), late leaf spot (LLS) and tomato spotted wilt virus (TSWV) in groundnut (Agarwal *et al*., 2018); environmental adaptation in Chinese soya bean landraces (Li *et al*., 2020b); agronomic‐related traits in Lima bean (Garcia *et al*., 2021); and days to flowering and leaf shape in pigeon pea (Singh *et al*., 2022).

Here, a high‐quality chromosome‐scale genome for *V. unguiculata* subsp. *sesquipedalis* was generated by combining high‐fidelity (HiFi) long‐read sequencing with a Hi‐C interaction map. Genome comparison between the subsp. *sesquipedalis* and *unguiculata* revealed extensive genomic variations, and these variations resulted in gene loss. A *V. unguiculata* germplasm panel with 315 accessions (278 accessions were collected in this study, and 37 accessions were obtained from previous reports) was resequenced, and a genomic variation map was constructed. With further investigation of this genomic variation map, population differentiation and selection signals in genome were identified. Two traits, pod length (PL) and pod width (PW), were observed for these 278 accessions, and a GWAS was performed for both traits. The genetic basis of the two traits was uncovered, and some candidate genes for the regulation of variations in the two traits were also predicted. We provide novel insights into the population differentiation and genetic basis of key agricultural traits in *V. unguiculata*.

## Results

### A high‐quality chromosome‐scale genome for *V. unguiculata* subsp. *sesquipedalis*

**Figure 1:**
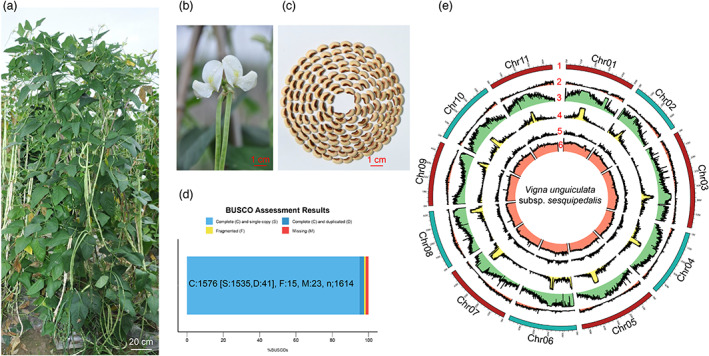
Genome assembly for *Vigna unguiculata* subsp. *sesquipedalis* line A147. (a) The *V. unguiculata* subsp. *sesquipedalis* line A147 was grown in the field. This photo was taken by the first author, Dr. Lei Pan. (b) Magnified images of flowers in a. (c) Magnified images of mature seeds in a. (d) BUSCO assessment of the assembled contigs of A147. (e) Features of the chromosome‐scale monoploid genome of A147. Numbers 1 to 6 represent scaffolds (pseudochromosomes), gene density, repeat content, Gypsy transposon content, *Copia* transposon content and GC content, respectively.

The elite breeding line A147 (also known as Xiabao II) of *V. unguiculata* subsp. *sesquipedalis* shows long pods (above 60 cm), white flowers and variegated seeds (Figure 1a–c). To assemble a high‐quality chromosome genome for A147, a total of 32.74 Gb clean HiFi long reads were generated (Table S1). The genome was then assembled by using two programs, HiFiAsm and Canu (Cheng *et al*., 2021; Koren *et al*., 2017), with default parameters. The HiFiAsm program generated 2141 contigs with a total size of 610 Mb and an N50 of 25.3 Mb (Table S2), while Canu generated 2143 contigs with a total size of 594 Mb and an N50 of 18.5 Mb (Table S2). Thereafter, a total of 70.3 Gb of Hi‐C clean reads were used to cluster both types of contigs into pseudochromosomes. The 594 Mb contigs generated by HiFiAsm could be perfectly clustered into 11 pseudochromosomes according to the Hi‐C matrix (Figure S1), while the 610 Mb contigs produced by Canu were not clustered into pseudochromosomes as expected. By manually correcting the pseudochromosomes with juicerbox (Durand *et al*., 2016), 539.4 Mb sequences were assigned to 11 pseudochromosomes in the final genome assembly of A147. These data revealed that approximately 91% of contigs could be assigned to chromosomes. With similarity to the chromosomes of *V. unguiculata subsp. unguiculata* IT97K, the 11 pseudochromosomes were assigned as Chr01 to Chr11, and this information is listed in Table S3. Chr03 showed the largest size, with 69.94 Mb, while Chr02 showed the smallest size of 37.80 Mb.

To assess the chromosome‐scale genome of A147, several strategies were applied. First, the pair‐end (PE) reads of our *V. unguiculata* germplasm panel, in which most lines are *V. unguiculata* subsp. *sesquipedalis*, were mapped onto the A147 genome, and most of these data showed a >98% mapping rate. Second, a total of 10 samples with approximately 75 Gb of RNA‐Seq data downloaded from SRA under project ID PRJNA763044 were also mapped onto the A147 genome by using a standardized RNA‐Seq analysis pipeline, and the average mapping rate was 95.64 ± 1.4% (Table S4). Both types of short reads showed a relatively high mapping rate, suggesting that the genome of A147 is of high quality. Third, BUSCO analysis showed 97.6% completeness, which also confirmed the high quality of the A147 chromosome‐scale genome assembly (Figure 1d). Fourth, the consensus quality value (QV) and completeness were also evaluated with Merquery pipelines for our genome assembly (Rhie *et al*., 2020). The QV and completeness were 35.89% and 95.61%, respectively, indicating the high accuracy of our assembly. Taken together, these data suggest that our genome assembly is of high quality and can be used in further analysis.

The 594 Mb A147 genome was first employed to identify repeat sequences. In total, 60.49% were annotated as repeat sequences (Table 1). The unclassified and LTR sequences accounted for the top two types of repeat sequences. An integration of *ab initio*, homology‐based, and transcriptome‐based gene prediction for A147 was also performed. In total, 27 439 genes encoding 41 725 proteins were predicted with an average length of 5621.4 and 1468.7 bp for genes and coding DNA sequences (CDSs) (Table 1), respectively. The gene features and repeat distributions are also illustrated in Figure 1e. All these genes were also functionally annotated with the Nr, TAIR, Swiss‐Prot, KEGG and InterPro databases. A total of 24 992 genes were examined, 89.3% of which could be annotated in at least one database (Table S5).

**Table 1:** Genome annotation of *V. unguiculata* line A147

Type	No. of elements	Length occupied (bp)	Percentage (%)
*Repeat sequence*			
LINEs	5458	144 238 806	0.35
LTR elements	181 064	2 092 293	23.93
DNA transposons	33 515	17 943 623	3.02
Rolling‐circles	4058	4 049 423	0.68
Unclassified	342 327	180 453 002	30.38
Simple repeats	160 881	10 494 566	1.77
Low complexity	31 498	2 111 047	0.36
Total	758 801	361 382 760	60.49

	No. of elements	Mean size (bp)
*Gene prediction*		
Gene	27 439	5621.4
Transcript	41 275	2087.1
CDS	41 275	1468.7
Exon	280 743	310.2
Intron	239 018	679.1

### Genome comparisons and evolution

**Figure 2:**
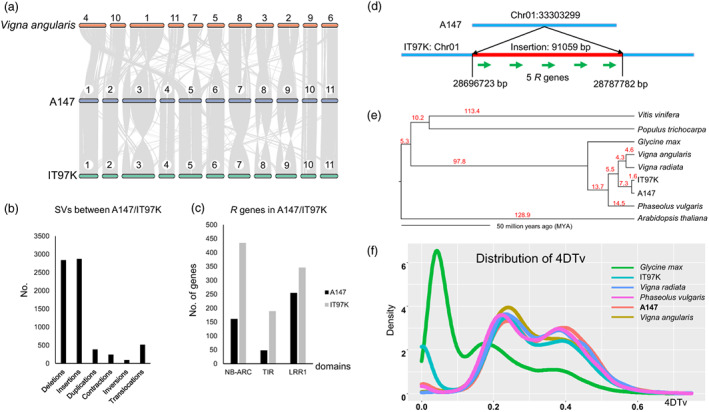
Genome comparisons between *Vigna unguiculata* subsp. *sesquipedalis* line A147 and its relatives. (a) Synteny comparisons of the chromosome‐scale genomes for the two pairs A147/IT97K and A147/*V. angularis*. Bars indicate chromosomes or scaffolds, while lines indicate synteny blocks between the two genomes. (b) The numbers of structural variations (SVs) between the genomes of A147 and IT97K. (c) The number of three types of resistance (R) genes with different domains in the genomes of A147 and IT97K. (d) R genes lost via large fragment deletion in the genome of IT97K Chr01: 33303299. The red bar indicates the lost DNA fragment, while green arrows indicate five lost *R* genes in the A147 genome. (e) Phylogenetic tree of nine plants, including *Vitis vinifera*, *Populus trichocarpa*, *Glycine max*, *V. angularis*, *V. radiata*, *Phaseolus vulgaris*, *Arabidopsis thaliana*, IT97K and A147. The red numbers indicate the divergence time. MYA indicates million years ago. (f) The density of fourfold synonymous (degenerative) third‐codon transversion (4DTv) values for six plants, including *G. max*, *V. angularis*, *V. radiata*, *P. vulgaris* IT97K and A147.

Previously, the high‐quality genome of *V. unguiculata* subsp. *unguiculata* line IT97K was also assembled and released (Lonardi *et al*., 2019). Therefore, a comparison of the two genomes was conducted. In total, there were 3866 collinear blocks with similarity >95% between the two genomes (Table S6). These collinear blocks accounted for 56.3% (334.5 Mb) of the 594 Mb A147 genomes. Of the 334.5 Mb collinear blocks, the longest and mean sizes were 81.5 kb and 91.0 kb, respectively. When illustrating the collinearity between A147 and IT97K in the genome‐scale view, seven chromosomes, Chr01, Chr02, Chr04, Chr05, Chr06, Chr10 and Chr11, showed perfect collinearity, while the remaining four chromosomes, Chr03, Chr07, Chr08 and Chr09, showed extensive rearrangement (Figure 2a). When comparing the genomes of A147 and IT97K with *V. angularis*, Chr03 of IT97K showed good collinearity with Chr01 in *V. angularis*; however, Chr07, Chr08 and Chr09 in A147 seemed to show better collinearity with the corresponding chromosomes in *V. angularis* (Figures 2a and S2). These results suggest that extensive genome rearrangement indeed existed in the 4 chromosomes in the species of *V. unguiculata*. The collinearity between A147 and *V. radiata* was also examined, and the results revealed much more chromosome rearrangement between these two species (Figure S3).

Structural variations (SVs) are >50 bp genomic variations among different genomes and include deletions, insertions, duplications, contractions, inversions and translocations. With comparisons of A147 with IT97K genomes, there were 7327 SVs, including 2837, 2871, 385, 243, 95 and 516 for deletions, insertions, duplications, contractions, inversions and translocations (Table S7 and Figure 2b), respectively. We focused on the two simple SVs, insertions and deletions, in the two genomes and found that these two types of SVs resulted in 1602 and 1880 nonredundant genes lost in IT97K and A147, respectively. According to the functions of these genes, it is very clear that a large number of *resistance* (*R*) genes were lost in the genome of A147 because of SV deletions. A comprehensive genome‐wide identification of *R* genes in these two genomes was performed. There were 161, 48 and 255 genes in the three types of *R* genes with NB‐ARC, TIR and LRR1 domains in the A147 genome (Figure 2c), respectively, while there were 435, 189 and 346 *R* genes with NB‐ARC, TIR and LRR1 domains in the IT97K genome (Figure 2c), respectively. These data indicated that the total numbers of *R* genes in A147 and IT97K are approximately 255 and 435, respectively, and there were 180 more *R* genes in the genome of IT97K. A total of 138 *R* genes were lost in A147 due to SV deletions (Tables S8 and S9). This result also suggested that approximately 77% of *R* genes in A147 were lost, resulting from SV deletion. According to the comparisons of A147 with IT97K, it was very easy to find the SV deletions that harbour *R* genes. For example, a 91 059 bp deletion in Chr01:33303299 of A147 (corresponding to Chr01:28696723‐28787782 of the IT97K genome) is illustrated in Figure 2d. Within this SV deletion, five *R* genes were lost. Taken together, our data suggested that SVs between A147 and IT97K resulted in extensive variations in gene contents and that these variations might play major roles in the differentiation between the two subspecies in *V. unguiculata*. Additionally, although we could not exclude some variations identified between A147 and IT97K that were attributed to different strategies for genome assembly and annotation, most of these variations would be reliable because both genome assemblies are of high quality.

The phylogeny constructed by using single‐copy genes among eight plants, including *Vitis vinifera*, *Populus trichocarpa*, *Glycine max*, *V. angularis*, *V. radiata*, *Phaseolus vulgaris*, *Arabidopsis thaliana*, IT97K and A147, suggested that the divergence between IT97K and A147 occurred recently (Figure 2e). The *V. unguiculata* species diverged from *V. angularis* and *V. radiata* 8.9 MYA (Figure 2d), while the *Vigna* genus diverged from the *Phaseolus* genus 14.5 MYA (Figure 2e). To investigate whole genome duplications in A147, the fourfold synonymous (degenerative) third‐codon transversion (4DTv) values of six plants, including *G. max, V. angularis*, *V. radiata, P. vulgaris* IT97K and A147, in the family of legumes were calculated. The distribution of these 4DTv values showed that there were two peaks in five plants, excluding *G. max*. The first peak occurred at approximately 0.38, while the other peak occurred at approximately 0.24 (Figure 2f). In previous studies, several plants in the spurge family (Euphorbiaceae) and *V. vinifera* also showed peaks of 4DTv at approximately 0.38, and this peak was regarded as a WGD event in the common ancestor of these plants (Luo *et al*., 2022). These data also indicated that peaks of 4DTv at approximately 0.38 is not specific to the family of legumes. The second peak of 4DTv at approximately 0.24 was found in *V. angularis*, *V. radiata, P. vulgaris* IT97K and A147 but not in *G. max*. However, an obvious peak of 4DTv at approximately 0.18 was found only in *G. max*. These data suggested that a second WGD occurred in the family of legumes; however, the time of the second WGD was different among genera. Additionally, there was also a third peak of 4DTv close to 0 for IT97K, and we speculated that it could be attributed to mistakes in its gene prediction.

### Genomic variants for a panel of widely used *Vigna unguiculata* germplasms and their population structure

A total of 278 widely used *V. unguiculata* germplasms in China were collected and used for the investigation of genomic variants (Table S10). An average of 10× coverage resequencing data was generated for each accession. Additionally, the resequencing data of 37 *V. unguiculata* accessions used in a previous study were also collected in our analysis (Lonardi *et al*., 2019). In total, 315 accessions were used in our analysis. Of these 315 accessions, there were 245 and 70 lines for subsp. *sesquipedalis* and subsp. *unguiculata*, respectively. All clean pair‐end (PE) reads were mapped onto the high‐quality chromosome‐scale genome of A147, and genomic variants, including SNPs and InDels, were identified. After quality filtering, a total of 17 831 792 genomic variants were identified for these 315 *V. unguiculata* accessions. The top three locations for these 17 831 792 genomic variants were intergenic 13 747 834 (53.939%), upstream of genes 4 273 944 (16.679%) and downstream of gene 3 923 595 (15.394%) (Table S11). A total of 46 569 (0.183%) genomic variants resulted in a strongly modified effect on genes (Table S11).

**Figure 3:**
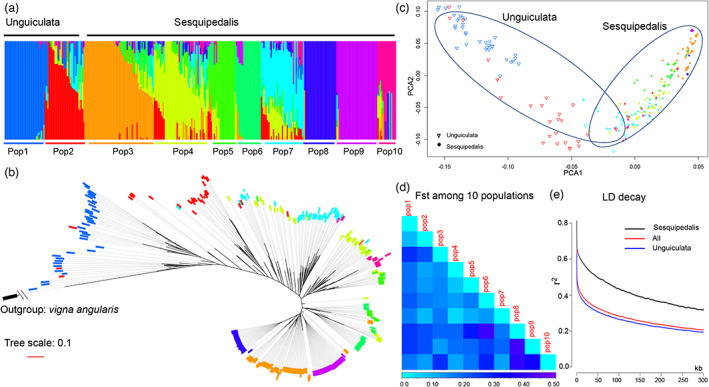
Population analyses of the *Vigna unguiculata* germplasm. (a) Population structure of the 315 accessions of *V. unguiculata* germplasm. Different colours indicate different pedigrees. (b) Phylogenetic tree of the *V. unguiculata* germplasm constructed by resequencing genomic data. *Vigna angularis* was used as an outgroup. The colours indicate different subpopulations assigned in (a). (c) Principal component analysis (PCA) for all *V. unguiculata* germplasms. The colours indicate different subpopulations assigned in (a), while triangles and dots indicate *V. unguiculata* subsp. *unguiculata* and *sesquipedalis*, respectively. PCA1 and PCA2 are dotted in this figure. (d) Genetic differentiation (*F*_st_) between each pair of subpopulations assigned in a. (e) Linkage disequilibrium (LD) decay for all the *V. unguiculata* germplasm, *V. unguiculata* subsp. *unguiculata* and *V. unguiculata* subsp. *sesquipedali*s.

Using the above high‐quality genomic variants, the population structure of these 315 *V. unguiculata* accessions was predicted by using admixture software (Alexander *et al*., 2009). The delta *K* values indicated that there were 10 subpopulations among these 315 accessions (Figure S4). These ten subpopulations were labelled Pop1 to Pop10 (Figure 3a). Clearly, all the subsp. *unguiculata* accessions were assigned to Pop1 and Pop2, while all the subsp. *sesquipedalis* accessions were assigned to Pop3 to Pop10. Of the two subpopulations in subsp. *unguiculata*, most accessions in Pop1 showed a unique pedigree, while Pop2 showed an admixed pedigree with a number of subpopulations in subsp. *sesquipedalis*. In the other eight subpopulations in subsp. *sesquipedalis*, most of the accessions in Pop8 and Pop9 showed unique pedigrees. Some accessions in Pop4 and Pop7 showed admixed pedigrees from subsp. *unguiculata*, while the other subpopulations showed only admixed pedigrees from the other subpopulations in subsp. *sesquipedalis*. These data suggested that there was hybridization between subsp. *sesquipedalis* and *unguiculata*. The numbers of accessions harboured in both pedigrees from subsp. *sesquipedalis and unguiculata* were not large, which also suggested that the hybridization between subsp. *sesquipedalis and unguiculata* was not extensive. PCA and phylogenetic analyses using these 17 831 792 genomic variants were also conducted (Figure 3b,c). In the PCA plot, approximately 20 accessions were placed in the overlapping distributions for subsp. *sesquipedalis and unguiculata*, while the other accessions showed clearly different distributions between the two subspecies (Figure 3b). The phylogeny constructed for all 315 accessions plus the outgroup *V. angularis* showed a classification of all the accessions very similar to that obtained in the population structure analysis (Figure 3c). Therefore, the results of all three analyses suggested that hybridization was not extensive between subsp. *sesquipedalis* and *unguiculata*. Moreover, the large number of subpopulations also suggested extensive divergence occurred in *V. unguiculata*, especially in subsp. *sesquipedalis*.

The phylogenetic analysis also revealed that all accessions of subsp. *unguiculata* were closer to the outgroup of *V. angularis* than subsp. *sesquipedalis*. This result suggested that subsp. *unguiculata* diverged from the ancestor of the genus *Vigna* first, and subsp. *sesquipedalis* originated from subsp. *unguiculata*. This result is highly consistent with a previous study (Herniter *et al*., 2020). To investigate the genetic differentiation among the 10 subpopulations, the *F*_st_ values between each pair were calculated (Figure 3d). Pop6‐Pop8 and Pop8‐Pop9 showed the highest *F*_st_ values, which were close to 0.5, indicating that there was relatively low gene flow between accessions in both pairs, although they belong to the same subspecies. The two subpopulations Pop1 and Pop2 that belong to subsp. *unguiculata* showed moderate genetic differentiation from all subpopulations in subsp. *sesquipedalis*. Interestingly, some pairs that include one subpopulation in subsp. *sesquipedalis* and another subpopulation in subsp. *unguiculata* showed very low *F*_st_ values, e.g., Pop2‐Pop8 and Pop2‐Pop10. This result suggested that the gene flow between the two subspecies is not always weaker than the gene flow within the same subspecies. The LD decay of all 315 accessions, the subsp. *sesquipedalis* and *unguiculata* was also calculated (Figure 3e). Clearly, LD decays faster in subsp. *unguiculata* than in *sesquipedalis* (Figure 3e). The overall LD decay distance (*r* ^2^ = 0.20) for all 315 accessions was approximately 300 kb. The nucleotide diversity (Pi) for subsp. *unguiculata* and *sesquipedalis* were 0.276 ± 0.158 and 0.118 ± 0.157, respectively. These data revealed that the nucleotide diversity is much higher in subsp. *unguiculata* than in *sesquipedalis*, which is also consistent with the LD decay analysis.

Collectively, the above analyses showed that there were a large number of genomic variants in the *V. unguiculata* germplasm panel. Population structure analysis supported that a large number of subpopulations were in this germplasm panel, especially in subsp. *sesquipedalis*. The data also supported that subsp. *unguiculata* diverged first and subsp. *sesquipedalis* originated from subsp. *unguiculata*. The nucleotide diversity is much higher in subsp. *unguiculata* than in *sesquipedalis*, and this phenomenon might result in LD decay faster than that in subsp. *unguiculata*.

### Genomic differentiation between subsp. *sesquipedalis* and *unguiculata*

**Figure 4:**
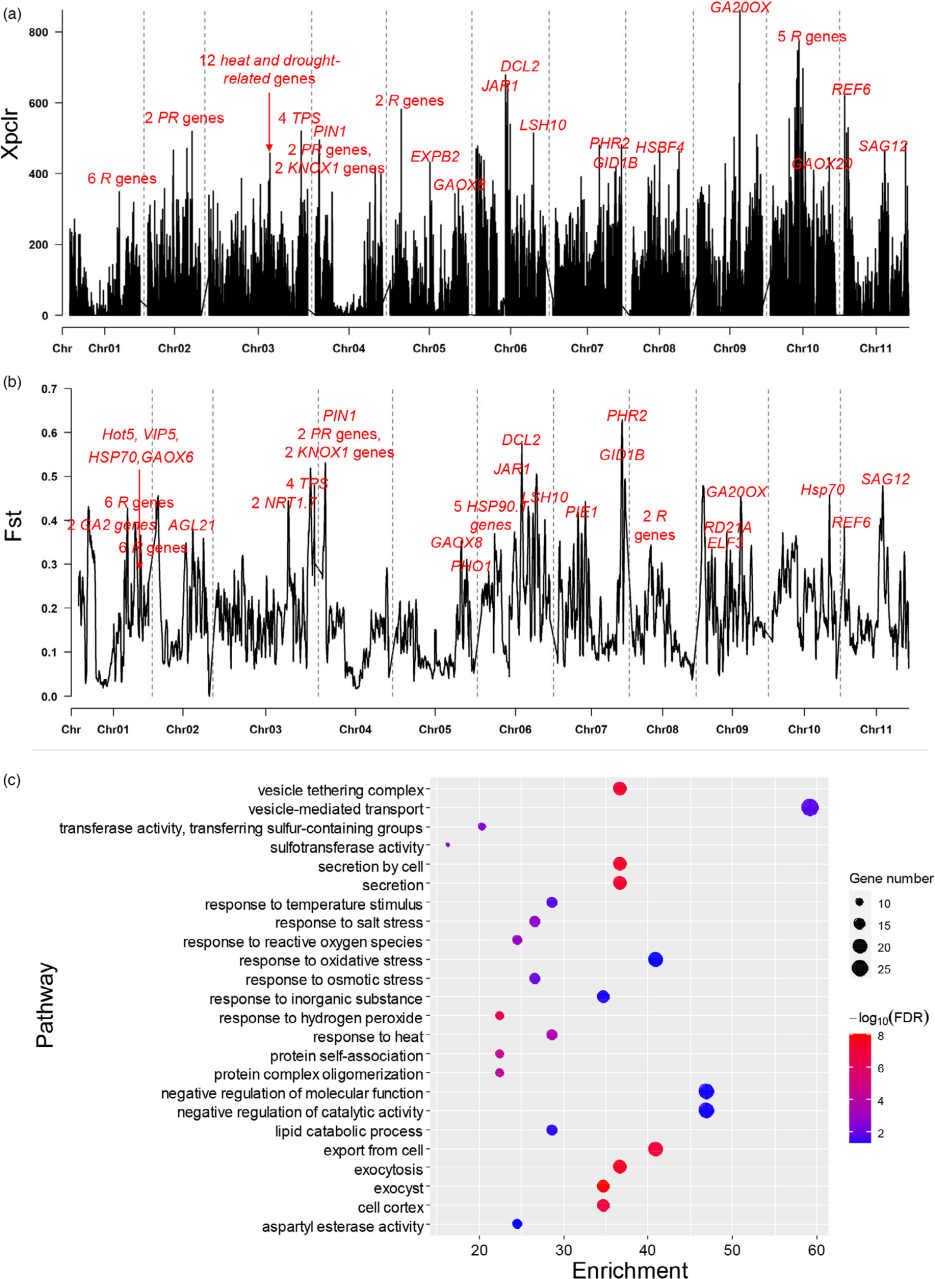
Genetic differentiation analysis between *Vigna unguiculata* subsp. *unguiculata* and *sesquipedali*s. (a) The sliding‐window cross‐population composite likelihood ratio (XP‐CLR) on 11 chromosomes for the two subpopulations. (b) The sliding‐window *F*_st_ on 11 chromosomes for the two subpopulations. Genes with known functions related to environmental adaptation in the genomic regions with the peak values are marked in red in both a and b. (c). Functional enrichment of genes located in both the top 5% XP‐CLR and *F*_st_ genomic regions.

Subsp. *unguiculata* is mainly distributed in Africa, while subsp. *sesquipedalis* is mainly planted in East Asia. Distinct growth environments in these two areas might shape different genomic characteristics for these two subspecies. To prove this prediction, two parameters, the sliding window of *F*_st_ and the cross‐population composite likelihood ratio (XP‐CLR) on 11 chromosomes for the two subpopulations, were calculated. The plots for these two parameters are illustrated in Figure 4a,b for *F*_st_ and XP‐CLR, respectively. When gaining insight into the functions of genes located in peak regions on each chromosome, a number of genes clearly showed functions related to biotic and abiotic resistance. For example, *R* genes, *pathogen resistance* (*PR*) genes and heat‐ and drought‐related genes could be found on the peaks of both XP‐CLR and *F*_st_ (Figure 4a,b). Interestingly, some genes related to auxin and gibberellin transport or biosynthesis, e.g., *PIN1*, *GA20OX* and *GAOX8*, were also located on the peaks of both XP‐CLR and *F*_st_ (Figure 4a,b). A comprehensive analysis of variations in *PIN1* (*evm.TU.Chr04.258*) revealed 47 genomic variants that formed two haplotypes (Hap1 and Hap2) and three genotypes (Hap1/Hap1, Hap1/Hap2 and Hap2/Hap2) in the 315 accessions (Table S13). There were 263, 1 and 51 accessions for genotypes Hap1/Hap1, Hap1/Hap2 and Hap2/Hap2 (Table S13), respectively. In the 245 accessions of subsp. *sesquipedalis*, there were 244 Hap1/Hap1 and one Hap1/Hap2, respectively; while there were 19 and 51 Hap1/Hap1 and Hap2/Hap2 in subsp. *unguiculata*. These data clearly suggested that genomic variants in *PIN1* gene showed significant differentiation between subsp. *sesquipedalis* and *unguiculata* (chi‐square analysis, *P* value < 0.05). The average PL of the three genotypes, Hap1/Hap1, Hap1/Hap2 and Hap2/Hap2, were 54.02 ± 12.03, 50.65 and 21.35 ± 10.98 cm (Table S13), respectively. Accessions with Hap1 showed significantly higher average PL than Hap2 (*P* value < 0.05). These data suggested that genomic variants in the *PIN1* gene showed a significant association with PL variations although the corresponding genomic region did not show a significant association in further GWAS. These data further suggested that genomic differentiation between subsp. *sesquipedalis* and *unguiculata* might be attributed to a number of trait variations between these two subspecies.

To comprehensively investigate the genetic differentiation between the two subspecies, the top 5% XP‐CLR and *F*_st_ genomic regions were identified and considered highly differentiated regions. A total of 1347 genes were located in both the top 5% XP‐CLR and *F*_st_ genomic regions (Table S14). A functional analysis of these 1347 genes showed that a number of GO terms related to environmental adaptation, including response to temperature stimulus, response to salt stress, response to reactive oxygen species, response to oxidative stress, response to inorganic substance, response to hydrogen peroxide and response to heat, were enriched. All these GO terms are related to the regulation of high temperature and salt stress. The subsp. *sesquipedalis* is mainly grown in Asia, while subsp. *unguiculata* is mainly grown in Africa. Differences in soil temperatures and salinities are the two major differences between the two growth conditions. Therefore, genes located in the highly differentiated genomic regions between subsp. *sesquipedalis* and *unguiculata* might be responsible for environmental adaptations.

### Artificial selections of pod length (PL)

**Figure 5:**
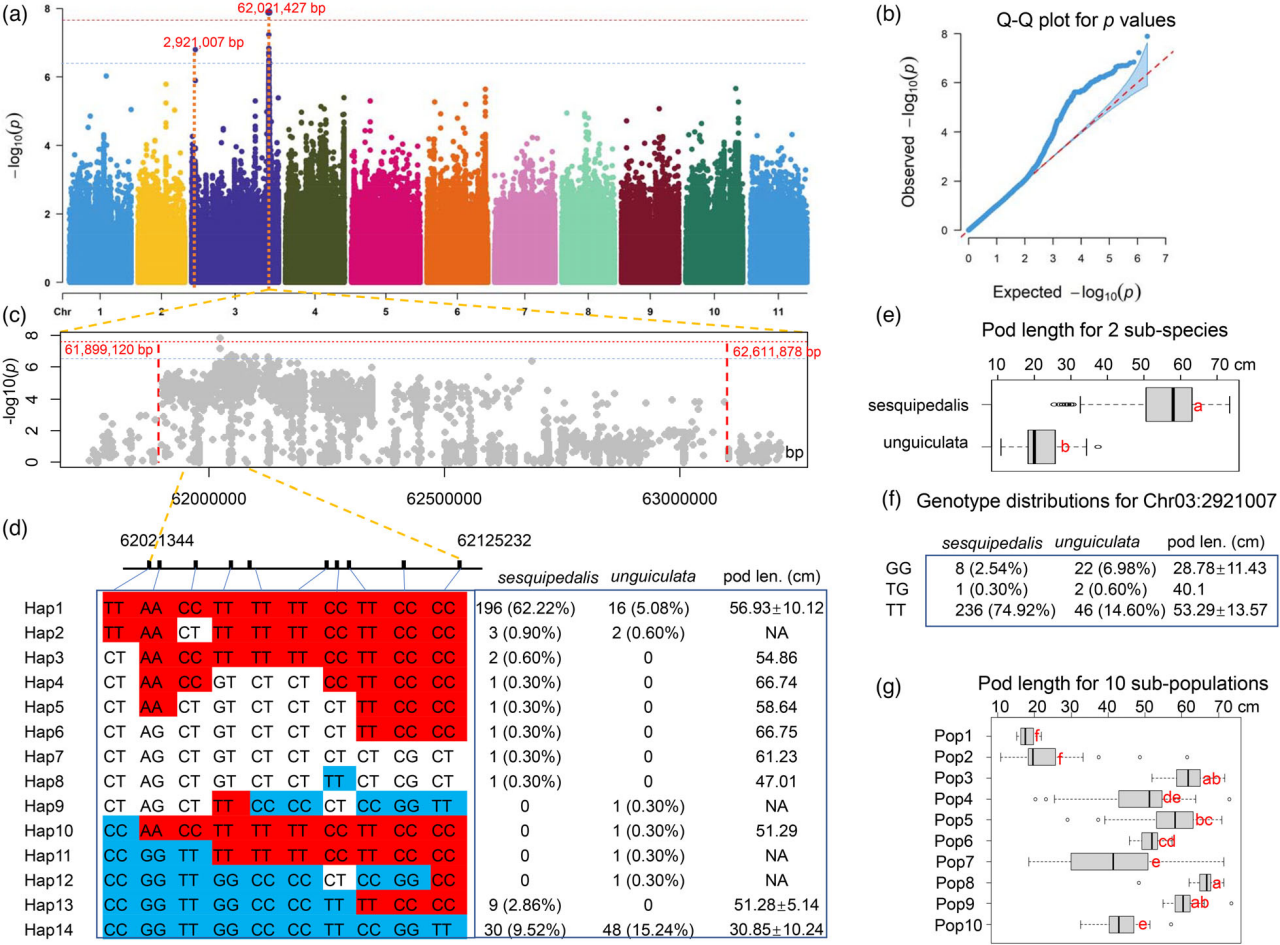
Genome‐wide association study (GWAS) of pod length (PL) variations in *V. unguiculata* germplasm and haplotype analyses of the QTLs. (a) Manhattan plot for the GWAS results of PL. The lead SNPs for the two QTLs are highlighted with their genomic positions. Two thresholds are outlined with red dots (2.31 × 10^−7^, 0.05/total tag SNP number) and blue dots (4.63 × 10^−8^, 0.01/total tag SNP number). (b) Quantile–quantile Plot for the *P* values in a. (c) The magnified genomic regions for the QTL at Chr03:61899120‐62027427. (d) The locations of significant SNPs in the QTL at Chr03:61899120‐62027427 and their haplotypes. The combinations of these significant SNPs are listed in the left panel, while the PL values for the corresponding haplotypes were calculated. In the table of the right panel, numbers indicate the plant numbers, while the percentage indicates the percentage of this number in all 315 accessions. (e) PL values for the *V. unguiculata* subsp. *unguiculata* and *sesquipedalis*. (f) Genotype distributions for the PL QTL at Chr03:2921007. The table lists data similar to those in the right panel of d. (g) Comparisons of PL values among the 10 subpopulations that were calculated in the prediction of population structure.

In Africa, people prefer to consume seeds of *V. unguiculata*, while pods are mainly consumed in Asia. Thus, PL is one of the most important traits in *V. unguiculata*. To investigate the genetic regulation of PL, this trait in the 278 germplasm lines was recorded in the field for five successive years, 2017–2021. A GWAS was performed by combining the genotypes and phenotypes for these 278 lines with a linear mixed model (LMM) model implemented in GEMMA software (Zhou and Stephens, 2014). A total of 3 429 792 high‐quality SNPs/indels were screened and used as genotypic data for GWAS. The tag SNP that represents variants in a region of the genome with high linkage disequilibrium was also identified, and there were 215 650 tag SNPs for the 278 lines. The genome‐wide significance thresholds of *P* values were set to 2.31 × 10^−7^ (0.05/total tag SNP number) and 4.63 × 10^−8^ (0.01/total tag SNP number) after Bonferroni correction. The Manhattan plot revealed that two QTLs showed an association with PL (Figure 5a). The Q–Q plot suggested that these QTLs were of high confidence (Figure 5b). Both associations are located on chromosome 3. Of these two QTLs, there were only significant SNPs above the threshold for the first QTL, and it was marked as Chr03:2921007, while the second QTL showed 10 SNPs above the threshold. Previously, some QTLs for PL were identified on chromosomes 3 and 8 by GWAS or linkage‐based QTL mapping (Lo *et al*., 2018; Xu *et al*., 2017); however, the locations of QTLs did not overlap with our results with a comprehensive comparison. The different QTL locations of PL in our study and previous studies might be attributed to the different plant materials used. Regarding the function of SNP Chr03:2921007, unfortunately, it was not located on any predicted genes. According to the *P* values of SNPs in the second QTL, it was easy to define its region as Chr03:61899120‐62027427 (Figure 5c). Within this QTL region, there are 36 predicted genes. However, it is difficult to select causal genes for this QTL according to their prediction functions (Table S15).

To see the selections of PL between subsp. *sesquipedalis* and *unguiculata*, the average PLs for these two subspecies were calculated. The average PLs for subsp. *sesquipedalis* and *unguiculata* were 55.87 ± 5.90 (*n* = 236) and 22.00 ± 5.90 (*n* = 30) cm (Figure 5d), respectively. The PL of subsp. *sesquipedalis* was much higher than that of *unguiculata* (*P* < 0.05). To determine the genetic variation between subsp. *sesquipedalis* and *unguiculata* in the two PL QTLs, haplotype analysis of the ten significant SNPs in Chr03:61899120‐62027427 was performed, and a total of fourteen haplotypes that were assigned as Hap1 to Hap14 were calculated (Figure 5e). Only four haplotypes, Hap1, Hap13 and Hap14, harboured more than five accessions. According to the phenotypes of PL among these haplotypes, Hap14 was obviously consistent with a short PL, while Hap1, Hap3‐8, Hap10 and Hap13 were consistent with a long PL. There were no PL data for four haplotypes (Hap2, Hap9, Hap11, Hap12) (Figure 5d). Regarding the classification of two subspecies in the three haplotypes that harboured more than five accessions, Hap13 included only one subpopulation, while Hap1 and Hap14 included both subpopulations. Based on similar analysis, SNPs within the second QTL for PL could result in three haplotypes that were marked as GG, TG and TT (Figure 5f). Haplotype GG was consistent with a short PL, while TG and TT were consistent with a long PL. Similarly, no haplotype included only one subspecies, *sesquipedalis* or *unguiculata*. Taken together, all the above data revealed that although some haplotypes in QTLs Chr03:61899120‐62027427 and Chr03:2921007 showed an association with PL variations, we could still not clearly see how subsp. *unguiculata* in Africa with a short PL evolved to subsp. *sesquipedalis* with a long PL in Asia.

In the above analyses, pedigree admixture was observed in the *V. unguiculata* germplasm. A total of ten subpopulations were observed, and Pop1, Pop8 and Pop9 showed relatively pure pedigrees in both population structure and phylogenetic analyses. Pop1 could represent the subsp. *unguiculata*, while Pop8 and Pop9 could represent the subsp. *sesquipedalis*. Therefore, the average PL values were also calculated, and these three subpopulations showed relatively uniform PL values (Figure 5g). Thus, investigation of the haplotypes in these three subpopulations might enable us to address the above question. With a combination of haplotypes in PL QTLs of Chr03:61899120‐62027427 and Chr03:2921007 in Pop1, Pop8 and Pop9, a total of nine genotypes were combined, including Hap1/TT, Hap1/GG, Hap1/TG, Hap2/TT, Hap3/GG, Hap12/TT, Hap14/GG, Hap14/TG and Hap14/TT (the former is for Chr03:61899120‐62027427, and the latter is for Chr03:2921007; Table 2). There were 4, 1, 1, 2, 0, 1, 12, 1 and 13 accessions for these nine combined genotypes in Pop1, while almost all accessions in Pop8 (26 accessions) and Pop9 (33 accessions) showed the combined genotype of Hap1/TT, excluding only one accession in Pop8 with Hap3/GG (Table 2). These data clearly indicated that the genetic diversity was much lower in Pop8 and Pop9 than in Pop1 at these two PL QTLs. The parameters of *F*_st_ (Pop1/Pop8) and *F*_st_ (Pop1/Pop9) were also calculated. The data revealed that the QTL at Chr03:61899120‐62027427 was located in the genomic regions that had >top 5% *F*_st_ (Pop1/Pop8) and *F*_st_ (Pop1/Pop9) (Figures S5 and S6), while the QTL at Chr03:2921007 was located in the genomic regions that had >top 5% *F*_st_ (Pop1/Pop9) (Figure S6). Thus, all these data suggested that strong selection existed in these two QTL regions during evolution from subsp. *unguiculata* to *sesquipedalis*. Generally, the trait of PL is not involved in environmental adaptation; thus, these selections could be attributed to artificial selection. Therefore, our results showed a clear genomic imprint of artificial selection on the PL of *V. unguiculata*. Additionally, this result also indicated the enhanced regulation of PL variation by QTLs at Chr03:61899120‐62027427 and Chr03:2921007.

**Table 2:** Combination of haplotypes at the pod length (PL) QTLs Chr03:61899120‐62027427 and Chr03:2921007 in Pop1, Pop8 and Pop9

Haplotypes combination	Pop1	Pop8	Pop9
Hap1/TT	4	26	33
Hap1/GG	1	0	0
Hap1/TG	1	0	0
Hap2/TT	2	0	0
Hap3/GG	0	1	0
Hap12/TT	1	0	0
Hap14/GG	12	0	0
Hap14/TG	1	0	0
Hap14/TT	13	0	0

### Genome‐wide association study (GWAS) of pod width (PW)

**Figure 6:**
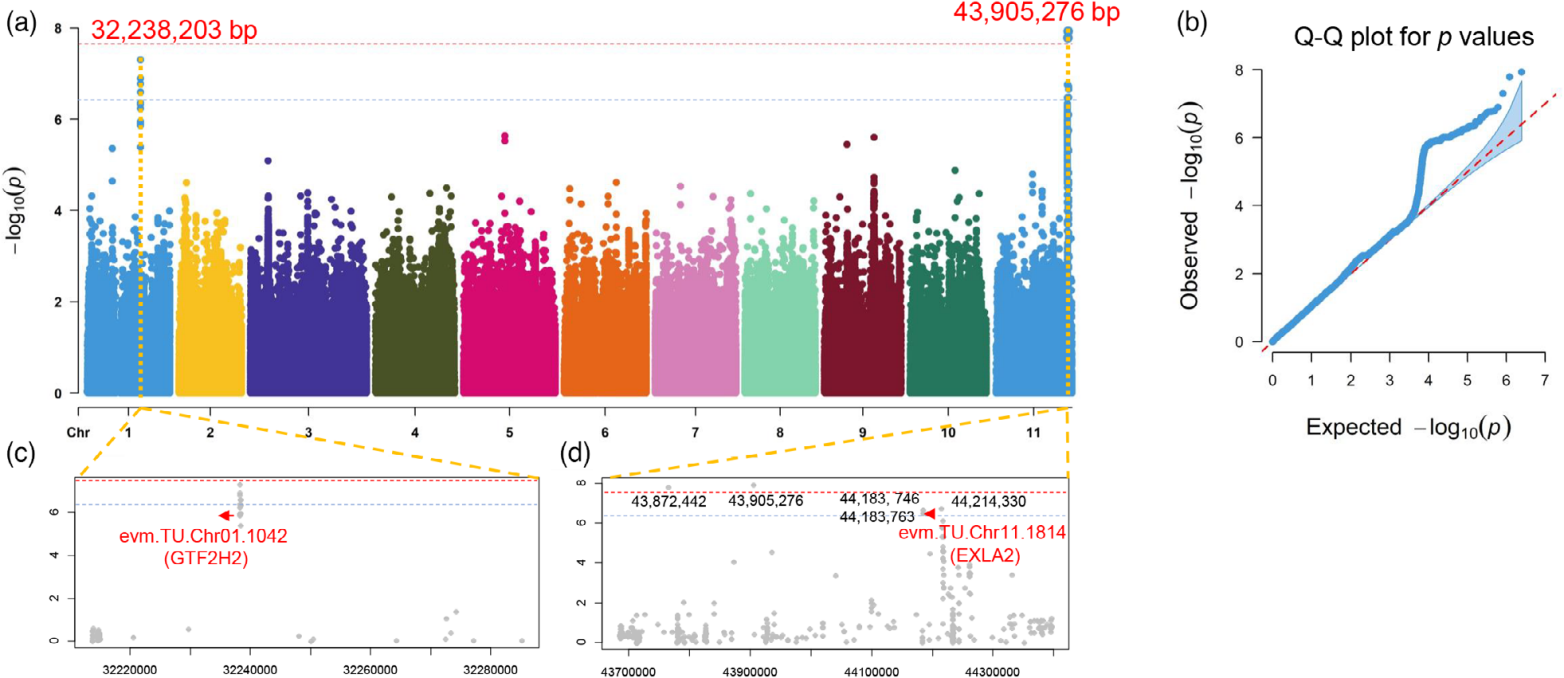
Genome‐wide association study (GWAS) of pod width (PW) variations in *Vigna unguiculata* germplasm. (a) Manhattan plot of the GWAS results of PW. The lead SNPs for the two QTLs are highlighted with their genomic positions. (b) Quantile–quantile plot for the *P* values in a. (c) The magnified genomic regions for the PW QTL at Chr01. (d) The magnified genomic regions for the PW QTL at Chr11. All other parameters are identical to those in Figure 5.

An additional important agricultural trait pod width (PW) was also recorded in the five successive years 2017–2021 for the 278 germplasm accessions. A comparison of the average PW between the subsp. *sesquipedalis* and *unguiculata* revealed that there was no significant difference (*P* = 0.39), suggesting that PW was not a challenge in selection. The GWAS showed that there were two major QTLs for PW located on Chr01 and Chr11 (Figure 6a). The Q–Q plot suggested that these QTLs were of high confidence (Figure 6b). For the QTL on Chr01, a total of 16 significant SNPs ranging from Chr01: 32238190 to Chr01: 32238353 showed much lower *P* values than their neighbouring SNPs; thus, this QTL was marked as Chr01: 32238190‐32238353. The 16 SNPs were not in any of the predicted genes; however, they were located 1220 bp upstream of the gene *evm.TU.Chr01.1042* (Figure 6c). A homology analysis of this gene suggested that it encoded a general transcription factor (TF) II H2 (GTF2H2). Because TFs are usually involved in the regulation of multiple biological processes, GTF2H2 would be the candidate gene for this QTL. For the other QTL on Chr11, significant SNPs ranged from Chr11: 43765142 to Chr11: 44217460; thus, it was marked as Chr11: 43765142‐44217460 (Figure 6a). In this QTL region, there were five significant SNPs, and none were located in the coding DNA sequence. Significant SNPs at Chr11:43872442, Chr11:44183746, Chr11:44183763 and Chr11: 44195129 were 43.0, 10.4, 2.6 and 2.6 kb from their nearest genes, respectively. The significant SNP Chr11:44214330 is 449 bp upstream of the gene *evm.TU.Chr11.1814*, which encodes expansin‐like A2 (EXLA2) (Figure 6d). In Arabidopsis, EXLA2 is involved in the response to cyclopentenone, plant‐type cell wall organization, unidimensional cell growth, and plant‐type cell wall loosening (Boron *et al*., 2015). Thus, it is highly possible that *V. unguiculata* EXLA2 is involved in the regulation of PW. Moreover, there were also a number of SNPs adjacent to Chr11:44214330 that showed a relatively low *P* value, although they did not reach the threshold of 2.31 × 10^−7^. Taken together, the two genes *evm.TU.Chr01.1042* (*GTF2H2*) and *evm.TU.Chr11.1814* (*EXLA2*) regulate the variation in PW in *V. unguiculata*.

## Discussion

### Valuable genomic resources for *Vigna unguiculata* genomics and breeding

In this study, we generated a high‐quality chromosome genome sequence for the subsp. *sesquipedalis* A147. Although there was a chromosome genome sequence for subsp. *unguiculata* in a previous report (Lonardi *et al*., 2019), large variations were observed between the two. Thus, the release of a high‐quality genome sequence for A147 is still necessary and valuable. Moreover, we also provided large genomic data for the 278 accessions that were widely used as breeding parents in China for *V. unguiculata*. These genomic data provided comprehensive genotypic information. According to this information, population structure and genetic relatedness were predicted. The classification of these 278 accessions into different subpopulations will guide us in selecting parents for making supercross combinations that will result in strong heterosis. Additionally, the phylogenetic analysis of all 315 accessions (including 37 accessions collected from a previous report) showed that *V. unguiculata* subsp. *unguiculata* is closer to the outgroup *V. angularis*. This result suggested that this subspecies was originally domesticated from its progenitor and that *V. unguiculata* subsp. *sesquipedalis* evolved from *V. unguiculata* subsp. *unguiculata*. Thus, our study is highly consistent with previous results (Fatokun *et al*., 2018; Lonardi *et al*., 2019; Xiong *et al*., 2016). Although we did not uncover new results regarding the origin of *V. unguiculata*, the comprehensive genomic data gave much greater support to previous reports.

Moreover, the QTLs of two traits of agricultural importance, PL and PW, were determined, and candidate genes for the regulation of variations in this trait were predicted. These results will help us develop molecular markers for both traits in their future molecular‐assisted selection (MAS). Also, we found a greater LD distance and lower genetic diversity in *V. unguiculata* subsp. *sesquipedalis* (Figure 3e). Thus, there is a need to break the strong LD and increase the genetic diversity in subsp. *sesquipedalis* to breed elite *V. unguiculata* lines. Taken together, these genomic data will greatly facilitate genomics and breeding programs in *V. unguiculata*. Additionally, more trait observations, e.g., disease resistance, efficiency of nutrition utilization and quality traits of pods, can be performed, and their underlying regulatory QTLs can be uncovered in the future.

### Natural and artificial selections shaped the features of the *Vigna unguiculata* genome

By comparing the two genomes of A147 and IT97K, we found a number of variations at the genomic level. A147 is regarded as a representative plant of *V. unguiculata* subsp. *sesquipedalis*, while IT97K is regarded as a representative plant of *V. unguiculata* subsp. *unguiculata*. The most prominent difference was the large variation in *R* gene numbers between the two genomes according to our comparisons. There were 180 more *R* genes in the genome of IT97K, and a total of 138 *R* genes were lost in A147 via SV deletions (Table S9). A number of previous studies pointed out that *V. unguiculata* was domesticated in Africa and then spread to other countries (Fatokun *et al*., 2018; Gbedevi *et al*., 2021; Herniter *et al*., 2020; Xiong *et al*., 2016). When *V. unguiculata* spread to China, the growth environment was totally different from its origin location. *R* genes are mainly involved in resistance to abiotic and biotic stresses. Thus, the loss of *R* genes in the A147 genome would result from growth adaptation for translocation from Africa to Asia for *V. unguiculata*. This result suggested that strong natural selection affected the evolution of *R* genes in the *V. unguiculata* genome.

Additional evidence also revealed natural selection in the *V. unguiculata* genome. When the genetic differentiation between *V. unguiculata* subsp. *sesquipedalis* and *unguiculata* was calculated with the two parameters *F*_*st*_ and XP‐CLR, and a number of genes were located in the differentiated genomic regions. The functional enrichment showed that most of these genes were involved in the regulation of environmental adaptations (Figure 4). Thus, this is additional evidence of natural selection affecting *V. unguiculata* genomes during the spread of this plant from Africa to China. This phenomenon is very common in a number of plants; e.g., natural selection shaped the genome of *Thlaspi arvense* and increased its adaptation to high elevations (Geng *et al*., 2021).

In addition to natural selection, artificial selection is also an important driver of genomic features. Many studies have uncovered how artificial selection affects genome features. In maize, long‐term artificial selection was found to affect ear numbers (Beissinger *et al*., 2014). In *Brassica napus*, different breeding preferences at different times have shaped different genomic features in the past 60 years (Hu *et al*., 2022; Wang *et al*., 2014). When *V. unguiculata* was introduced to China, people preferentially consumed its pods but not seeds, and *V. unguiculata* with edible pods was taxonomically classified as *V. unguiculata* subsp. *sesquipedalis*. Thus, the major breeding aim is different in China and Africa and would affect genomic features. Two major QTLs were identified for PL variations. Although we were not able to provide candidate genes for the two QTLs of PL, we found that genetic diversity at these two QTLs greatly decreased in *V. unguiculata* subsp. *sesquipedalis* population. Thus, strong artificial selection indeed affected *V. unguiculata* genomic regions that are responsible for the regulation of PL variations. In summary, natural and artificial selection jointly shaped the features of the *V. unguiculata* genome.

## Methods

### Plant materials, growth conditions and trait observations

The breeding line A147 (also known as Xiabao II), which is widely grown in China, was used as plant material for genome assembly. It was grown in the field in Hannan district, Wuhan, Hubei Province of China (N 114.08°, E 30.31°) with normal irrigation, and young seedlings were collected as samples for DNA extraction. Different tissues, including seedlings, young flowers, roots, young pods and young leaves, were collected for RNA preparation. A total of 278 germplasm accessions were collected and grown in the same field where A147 was grown for five successive years (2017–2021), including autumn 2017 (August 1st to November 15th), spring 2018 (April 18th to July 30th), autumn 2019 (August 1st to November 15th), spring 2021 (April 18th to July 30th) and autumn 2021 (August 1st to November 15th). The locations where these germplasm accessions were collected are listed in Table S10. All the germplasms were grown under normal field management. For trait observation, the length of the mature pods (PL) and the width of pods (PW) were measured from 2017 to 2021. At least five plants and 10 pods for each plant were measured.

### Genome assembly and assessment

For genome assembly, two types of genomic data were generated. High‐quality genomic DNA for A147 was isolated using the modified cetyltrimethylammonium bromide (CTAB) method (Porebski *et al*., 1997). These DNA samples were sequenced by the Single‐molecule real‐time (SMRT) Pacific Biosciences (PacBio) platform, and long high‐fidelity (HiFi) reads were obtained. The Hi‐C library for A147 was also constructed according to the manufacturer's protocol and sequenced with the Illumina HiSeq platform NovaSeq 6000 (Illumina, San Diego, CA). Two software programs, Canu and HiFiAsm (Cheng *et al*., 2021; Koren *et al*., 2017), were applied to assemble these long HiFi reads. The pseudochromosomes were constructed by 3d‐DNA pipelines with a “haploid” mode (Durand *et al*., 2016). The juicerbox pipeline was used to manually correct the pseudochromosomes (Durand *et al*., 2016). For genome assessment, the BUSCO pipeline was applied, and the embryophyta_odb10 database was downloaded on 2022‐01‐10 (Simao *et al*., 2015). Merquery pipelines were also applied to evaluate genome completeness and QV for our genome assembly with default parameters (Rhie *et al*., 2020). For RNA‐Seq mapping, raw RNA‐Seq data were downloaded from the SRA under project ID PRJNA763044, and these data were filtered by Trimmomatic with default parameters (Bolger *et al*., 2014). Reads were mapped onto the genome of A147 by HISAT2 with default parameters (Kim *et al*., 2019).

### Genome annotations

A combination of homology‐based and *de novo* approaches was applied for transposable element (TE) identification. Briefly, the TE database was first built with the software program RepeatModeler‐2.0.2a; then, the TEs were searched with the software program RepeatMasker v.4.0.5 (Benson, 1999). For gene prediction, *ab initio*, homology‐based and transcriptome‐based gene prediction strategies were applied for the repeat‐masked A147 genome. Briefly, homology‐based gene prediction was conducted by using the exonerate program, and the gene structures of five plants (Slater and Birney, 2005), including *A. thaliana*, *V. vinifera*, *O. sativa*, *P. trichocarpa* and IT97K, were used as homology references. The GlimmerHMM and AUGUSTUS pipelines were used for *ab initio* gene prediction (Majoros *et al*., 2004; Stanke *et al*., 2006). The long reads from RNA sequencing with the Iso‐Seq platform were used for transcriptome‐based gene prediction with Program to Assemble Spliced Alignments (PASA) version PASApipeline.v2.4.1 (Haas *et al*., 2003). All evidence for gene structures was integrated with EVidenceModeler (EVM, version 1.1.1) (Haas *et al*., 2008). Finally, these integrated generated gene structures were also updated with PASApipeline.v2.4.1. All parameters in these software programs were set as default.

All the predicted genes were then functionally annotated with the Swiss‐Prot, TrEMBL, TAIR and Nr databases by using blastp software. All these databases were accessed on 2021‐09‐01. Additionally, the functions of all genes were also annotated with automated assignment of human‐readable descriptions (AHRD) pipelines to assign the GO terms to each gene (https://github.com/groupschoof/AHRD). Functional enrichment was performed by using TBtools software (Chen *et al*., 2020).

### Genome comparisons

The collinearity between pairs among the *V. angularis*, A147 and IT97K genomes was performed by using the MUMMER and JCVI pipelines according to the manufacturer's instructions (https://github.com/tanghaibao/jcvi). Single‐copy genes were identified among eight plants, including *V. vinifera*, *P. trichocarpa*, *G. max*, *V. angularis*, *V. radiata*, *P. vulgaris*, *A. thaliana*, IT97K and A147, by using the OrthoFinder program (Emms and Kelly, 2019). Genomes of *V. vinifera*, *P. trichocarpa* and *A. thaliana* were downloaded from Phytozome on 2022‐05‐01 (https://phytozome‐next.jgi.doe.gov/), while genomes of *G. max*, *V. angularis*, *V. radiata*, *P. vulgaris* and IT97K were downloaded from the *V. unguiculata* database on 2022‐05‐01 (https://phytozome‐next.jgi.doe.gov/). The phylogenetic trees were constructed with the software RAxML (the model parameter was set as “PROTGAMMAAUTO”) (Stamatakis, 2014). The divergence times for these nine plants were estimated using the MCMCTree program, and they were calibrated with information supplied from the TimeTree website (http://www.timetree.org/) (Puttick, 2019). The fourfold synonymous (degenerative) third‐codon transversion (4DTv) values were calculated with the programs WGD (simple command line tools for the analysis of ancient whole‐genome duplications) and KaKs_Calculator2.0 (Wang *et al*., 2009).

### Genomic resequencing and variant calling

High‐quality DNA for the 278 germplasm accessions was sent for PE sequencing by using the Illumina HiSeq platform NovaSeq 6000 (Illumina, San Diego, CA). The PE reads for another 37 accessions were downloaded from the SRA database in GenBank (https://www.ncbi.nlm.nih.gov) (Table S10). The raw reads were filtered by using the Trimmomatic program with default parameters (Bolger *et al*., 2014). Clean reads were mapped onto the A147 genome with the “bwa mem” pipeline. Genomic variants were called with the standardized pipelines implemented in Genome Analysis Toolkit (GATK) (Dreval *et al*., 2021). SNPs/Indels were filtered with the parameters ‘QUAL <30.0 ¦¦ QD < 2.0 ¦¦ FS > 60.0 ¦¦ SOR > 4.0’. The functions of SNPs/InDels were predicted with the SnpEff program (Cingolani *et al*., 2012).

### Population analyses

Population structures for the 315 accessions were calculated with the program admixture (admixture_linux‐1.3.0), setting *K* from 1 to 20 (Alexander *et al*., 2009). Linkage disequilibrium (LD) was calculated and a phylogeny was constructed with the programs PopLDdecay and FastTree (Zhang *et al*., 2019). The PCA and *F*_st_ analyses among the ten subpopulations were performed with the PLINK program (Purcell *et al*., 2007). Genomic differentiation between subsp. *sesquipedalis* and *unguiculata* was predicted with two parameters, *F*_st_ and XP‐CLR. For *F*_st_ calculation, the program VCFtools was applied, and the *F*_st_‐window‐size and *F*_st_‐window‐step were set as 100 and 10 kb (Danecek *et al*., 2011), respectively. For the XP‐CLR calculation, the program XPCLR was applied, and the window size was set as 100 kb (Chen *et al*., 2010). All parameters except those mentioned above were set as default.

### Genome‐wide association study (GWAS)

The 278 *V. unguiculata* accessions were used as plant materials for the GWAS. The PL and PW were recorded in the field for five successive years. The best linear unbiased prediction (BLUP) values of PL and PW were used as trait data for the GWAS. The BLUP was conducted by using the “lme4” package implemented in R version 4.0.5 (https://www.r‐project.org/). Genomic variants, including SNPs and InDels, were used as genotypic data. Genomic variants showing a minor allele frequency (MAF) were excluded from further analysis. GEMMA (Genome‐wide Efficient Mixed Model Association algorithm) pipelines were used to perform the GWAS, setting the parameter LMM as 1 (Zhou and Stephens, 2014). The numbers of tag SNPs were determined with the PLINK program. The threshold for GWAS was set as 0.05/number of tag SNPs and 0.01/number of tag SNPs. A Manhattan plot for GWAS result visualization was constructed with the CMPlot program implemented in R software (https://www.r‐project.org/).

## Author contributions

Lei Pan, Minghui Liu, Yan Kang, Xiang Mei, Gege Hu, Chun Bao, Yu Zheng, Huixia Zhao and Chanyou Chen performed the field experiments. Nian Wang and Lei Pan conducted data analyses, manuscript writing, and editing. Lei Pan and Nian Wang supervised this project.

## Competing interests

The authors declare no conflict of interest.

## Supporting information


**Figure S1** Plot of the high‐throughput chromosome conformation capture (Hi‐C) matrix for the chromosome‐scale genome of *Vigna unguiculata* subsp. *sesquipedalis* line A147.
**Figure S2** Synteny comparisons of the chromosome‐scale genomes for two pairs of IT97K/A147 and IT97K/*V. angularis*.
**Figure S3** Synteny comparisons of the chromosome‐scale genomes for two pairs of A147/IT97K and A147/*V. radiata*.
**Figure S4** Determination of *K* values for population structure analysis.
**Figure S5** Genetic differentiation analysis between Pop1 and Pop8.
**Figure S6** Genetic differentiation analysis between Pop1 and Pop9.

**Table S1** Summary of sequence data used for genome assembly.
**Table S2** Summary of contigs assembled with different programs.
**Table S3** Summary of A147 pseudochromosomes.
**Table S4** Summary of the mapping rate for RNA‐Seq data.
**Table S5** Functional annotation of the A147 genome.
**Table S6** Genome comparisons of two subspecies in *Vigna*.
**Table S7** Structural variations (SVs) between the A147 and IT97K genomes.
**Table S8** Gene loss resulting from insertion and deletion of SVs in the IT97K genome.
**Table S9** Gene loss resulting from insertion and deletion of SVs in the IT97K genome.
**Table S10** The *V. unguiculata* germplasm and the classification of subspecies and subpopulations.
**Table S11** Summary of genomic variant effects by type and region.
**Table S12** Functions of genomic variants.
**Table S13** Analysis of genomic variants on *PIN1* (*evm.TU.Chr04.258*).
**Table S14** Genes located in highly differentiated regions.
**Table S15** Gene information in the pod length QTL region Chr03:61899120‐62027427.

## Data Availability

The whole genome sequence data and short reads for the 278 accessions have been deposited in the NCBI under accession number PRJNA890023. The genome sequence of A147 and its annotation files also can be obtained in our own website (http://tree‐bio.hzau.edu.cn/download/A147‐yard‐long‐bean).
