# Supplementary material for: Comprehensive genomic analyses of Vigna unguiculata provide insights into population differentiation and the genetic basis of key agricultural traits
Source: Plant Biotechnol J. 2023 Apr 4;21(7):1426–39. doi: 10.1111/pbi.14047 (PMC10281604; doi:10.1111/pbi.14047)
Supplement: Supplementary file 1 — Figure S1 Plot of the high‐throughput chromosome conformation capture (Hi‐C) matrix for the chromosome‐scale genome of Vigna unguiculata subsp. sesquipedalis line A147. Figure S2 Synteny comparisons of the chromosome‐scale genomes for two pairs of IT97K/A147 and IT97K/V. angularis. Figure S3 Synteny comparisons of the chromosome‐scale genomes for two pairs of A147/IT97K and A147/V. radiata. Figure S4 Determination of K values for population structure analysis. Figure S5 Genetic differentiation analysis between Pop1 and Pop8. Figure S6 Genetic differentiation analysis between Pop1 and Pop9. [file PBI-21-1426-s002.docx]

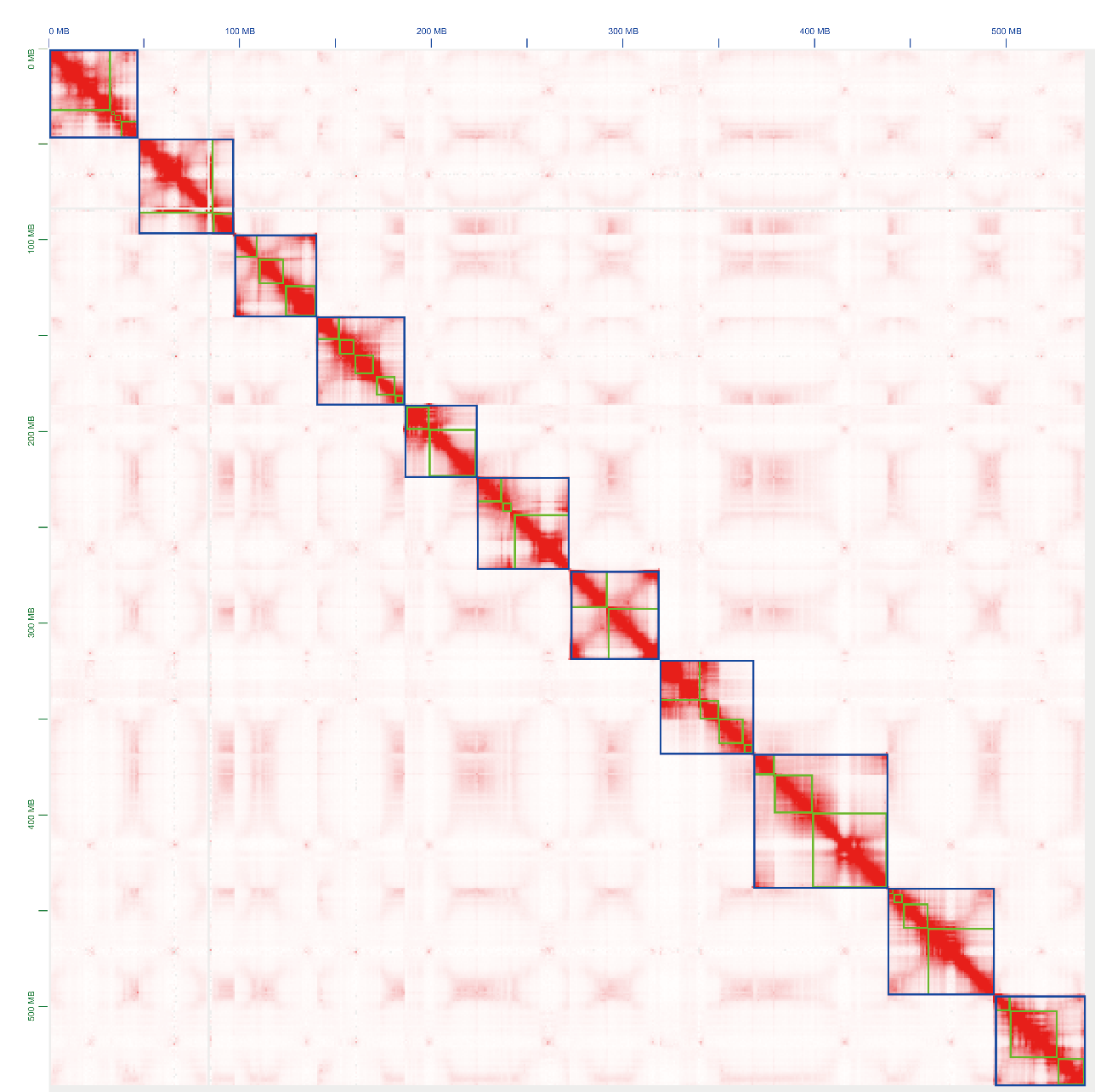


**Fig. S1.** Plot of High-throughput Chromosome Conformation Capture **(**Hi-C) matrix for the chromosome-scale genome of *Vigna unguiculata* subsp. *sesquipedalis* line A147

The chromosome orders for the x and y axis are the same and they are labeled in the bottom of this figure.


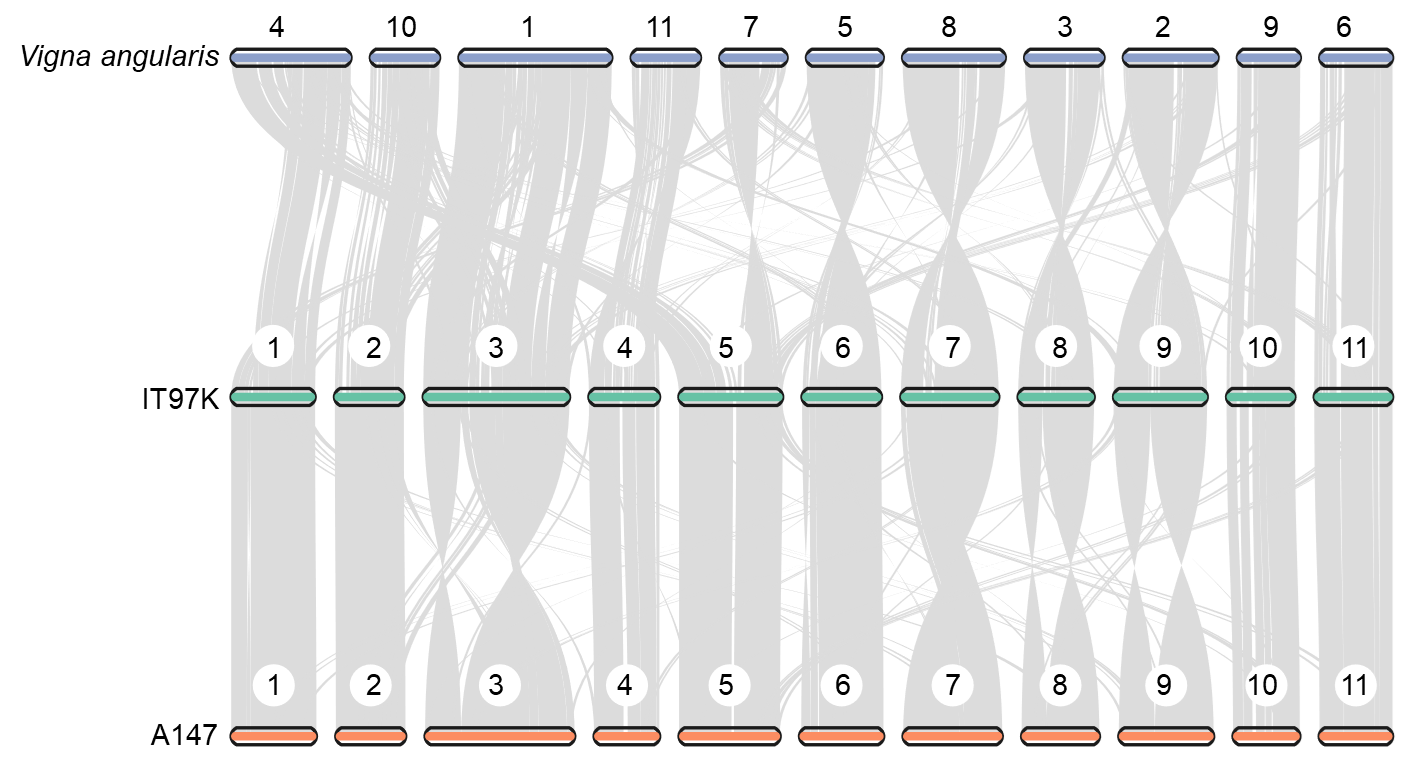


**Fig. S2.** Synteny comparisons of the chromosome-scale genomes for two pairs of IT97K/A147 and IT97K /*V. angularis*

Bars indicate chromosomes or scaffolds, while lines indicate synteny blocks between the two genomes.


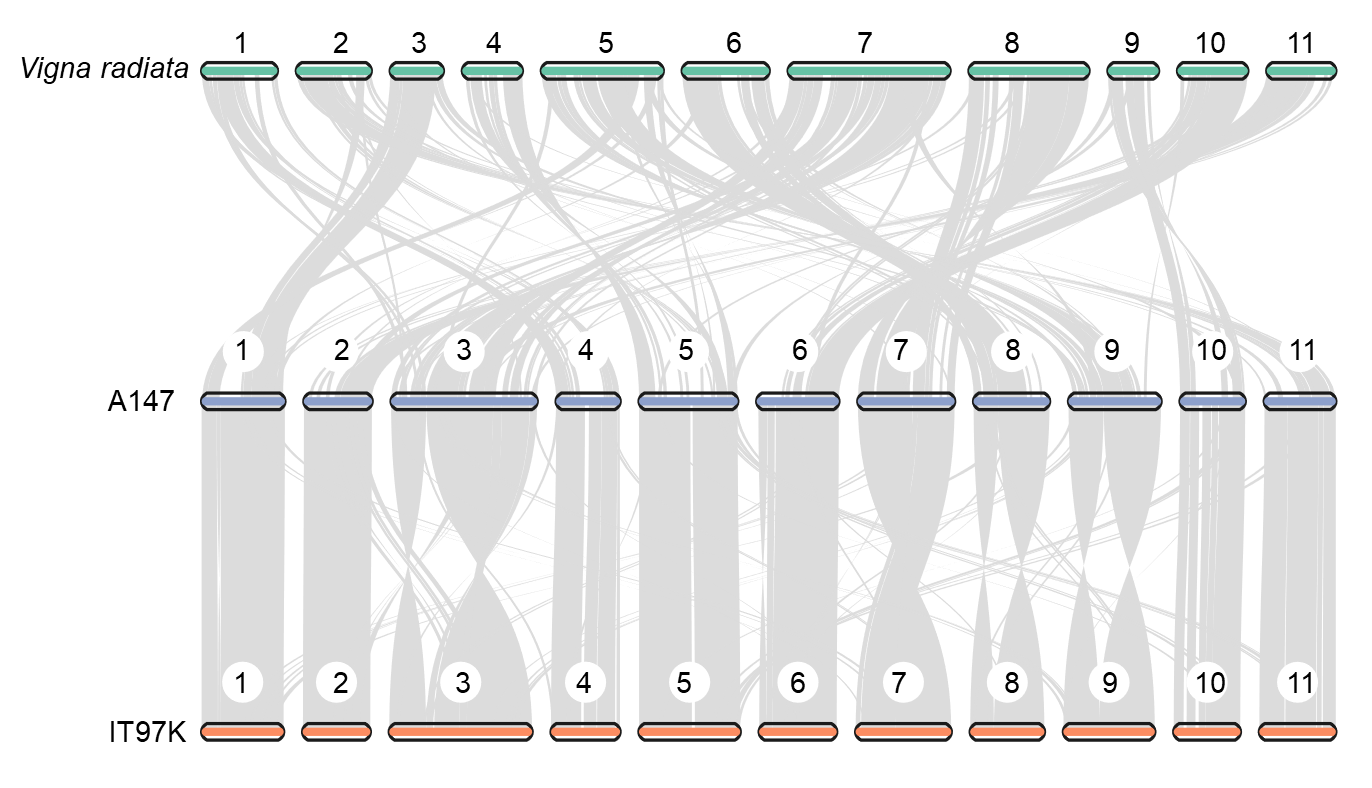


**Fig. S3.** Synteny comparisons of the chromosome-scale genomes for two pairs of A147/IT97K and A147 /*V. radiata*

Bars indicate chromosomes or scaffolds, while lines indicate synteny blocks between the two genomes.


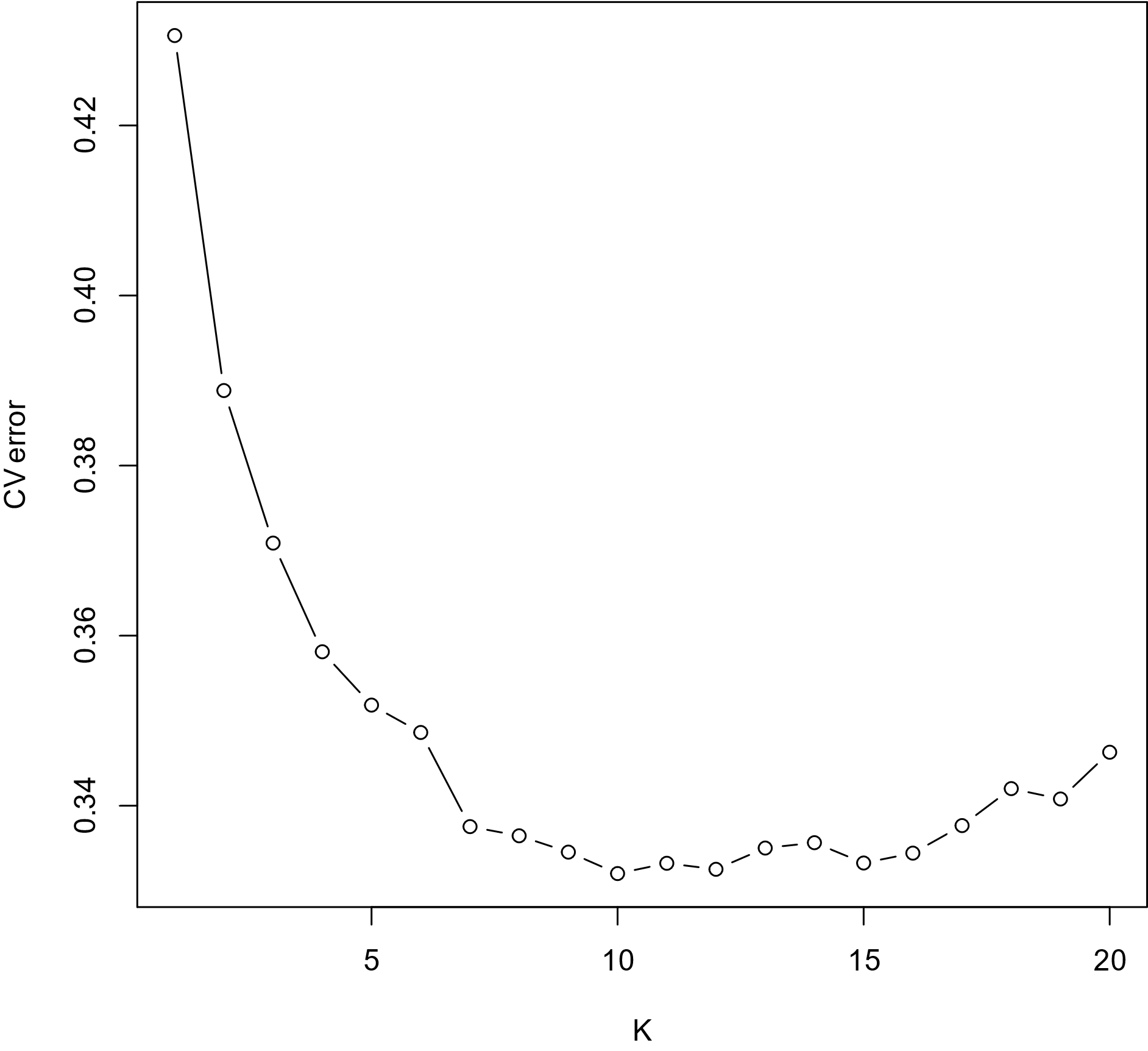


**Fig. S4.** The determination of K values for population structure analysis

The K values were set from 2 to 20 for calculation of delta K (CV error). When K equal to 10, the CV error showed the lowest value. Thus, K was selected as 10 and an optional 10 subpopulations in the 315 accessions of cowpea germplasm.


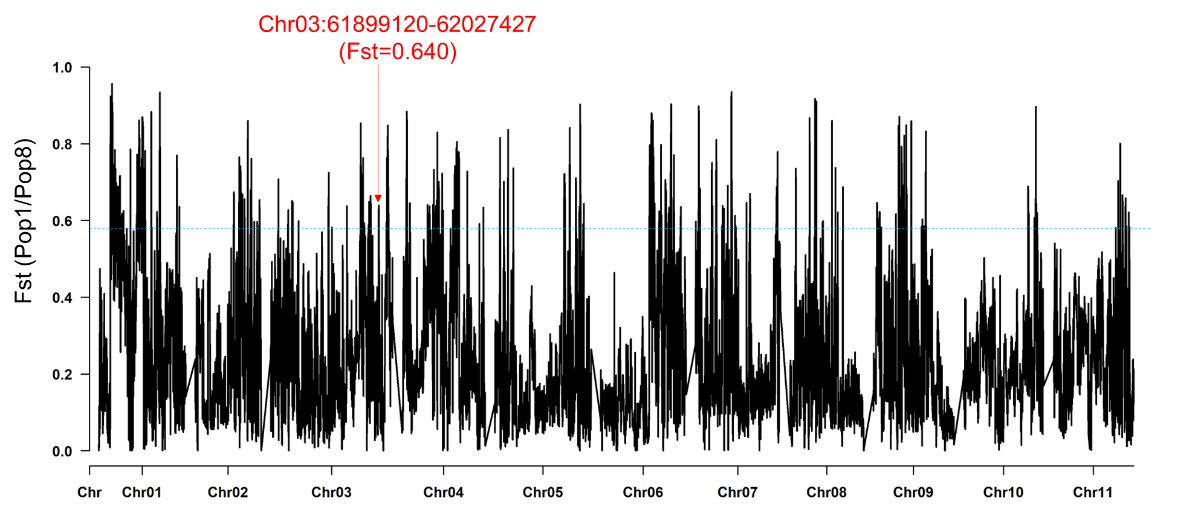


**Fig. S5.** Genetic differentiation analysis between the Pop1 and Pop8

Pop and Pop8 were determined with structure analysis. Their accession numbers were listed in Table S10. The blue dash line indicates the top 5% values of all Fst.


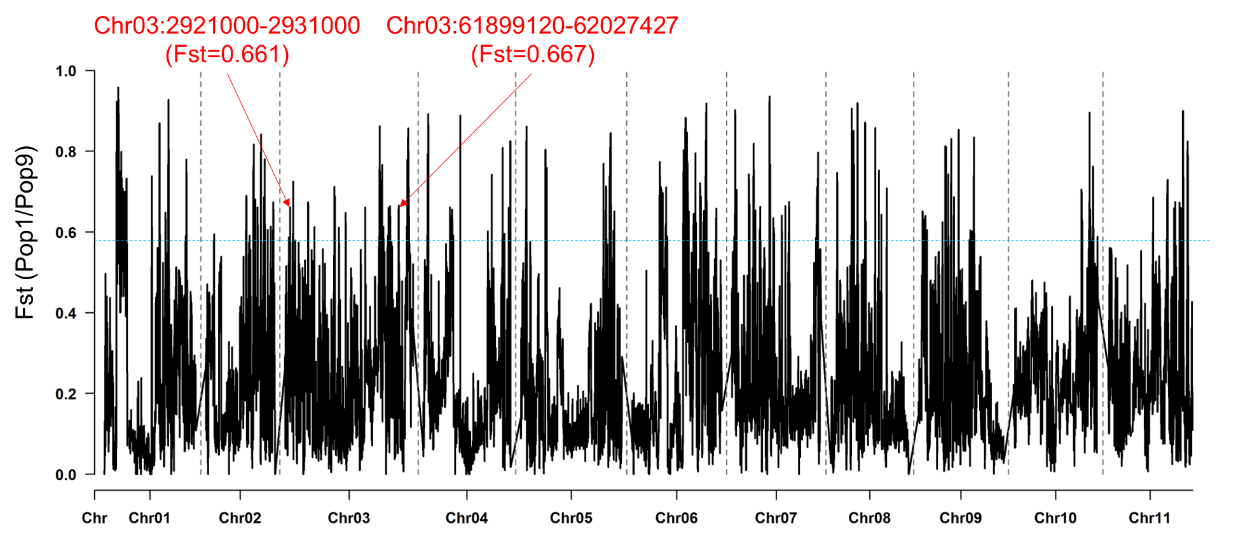


**Fig. S6.** Genetic differentiation analysis between the Pop1 and Pop9

Pop and Pop9 were determined with structure analysis. Their accession numbers were listed in Table S10. The blue dash line indicates the top 5% values of all Fst.
